# Laparoscopic resection rectopexy significantly affects preexisting urinary symptoms in female patients

**DOI:** 10.1007/s00384-022-04172-0

**Published:** 2022-05-06

**Authors:** Matthias Kraemer, Silvia Kraemer, Canan Ceran

**Affiliations:** Department of General and Visceral Surgery, Coloproctology, St. Barbara-Klinik, Hamm 59073 Germany

**Keywords:** Rectopexy, Urinary incontinence, Urinary frequency

## Abstract

**Purpose:**

It has previously been noted that following rectopexy, some patients report changes in urinary function. So far, not much is known about the extent of such changes. This study assesses the effects of laparoscopic rectopexy on urinary symptoms.

**Methods:**

Prospective observational study with 100 consecutive female patients indicated for laparoscopic resection rectopexy. Stated urinary symptoms, pre- and postoperative “International Consultation on Incontinence Questionnaire” (ICIQ), supplemented by a “quality of life “ (QoL) visual analogue scale, and residual urine measurements (RUM) were compared and correlated.

**Results:**

Postoperative QoL was significantly improved, irrespective of preexisting urinary symptoms. Twenty-four (24%) patients noticed improved urinary function. This corresponded with 42% of 45 patients who had positive preoperative ICIQ scores indicating preexisting urinary symptoms. Conversely, 14 (14%) patients noticed a postoperative increase of urinary complaints. The stated symptom change was only in part reflected by changes of the ICIQ scoring. Comparing ICIQ, 19 (19%) patients scored “better” postoperatively against 8% scoring worse; 5 of the 8 patients experienced “de novo” symptoms. The improved postoperative ICIQ scoring was highly significant. RUM did not sufficiently correlate to symptoms/ICIQ for any meaningful conclusion.

**Conclusions:**

Laparoscopic resection rectopexy had predominantly beneficial and to a lesser extent detrimental effects on urinary symptoms. Effects were highly significant; they were mainly noted in patients with preexisting urinary complaints. So far, it is not possible to predict such effects on an individual basis. It appears likely that similar effects may be found for most of the alternative operative procedures for the treatment of rectal prolapse. Without more factual knowledge and awareness about the extent of potential “collateral” effects of pelvic floor repair procedures, expert guidance of patients appears limited.

## Introduction

For many decades, the indication and technicalities of rectopexy for the treatment of occult and complete (“full thickness “) rectal prolapse have been debated in the literature. Despite this, the main issues involving indication per se and methodology (with or without resection; abdominal or perineal approach; the use of mesh or suture; ventral, circular, or dorsal rectal mobilisation etc.) have still not been conclusively resolved and continue to be discussed [1]. Among the numerous surgical options, laparoscopic resection rectopexy is one of the established procedures [1–5]. It is important to point out that this study does not contribute to the long-standing and in certain aspects cyclical debate surrounding the controversial issues.

Over the years, authors have occasionally noted changes in urinary function almost as by-product of their various studies on abdominal techniques of rectal prolapse repair [6–8]. Accordingly, it is also our experience that following laparoscopic resection rectopexy, patients quite commonly report changes in urinary frequency and urinary incontinence. However, so far not much is known about the extent of such changes. This study therefore aims to systematically assess and quantify the effects of laparoscopic rectopexy on urinary symptoms.

## Patients and methods

Included in this prospective observational study were 100 consecutive female patients indicated for laparoscopic resection rectopexy and willing to participate in the study. Patients were recruited between 4/2017 and 8/2019 via the coloproctological outpatient clinic run by our department. During the time of recruitment, seven patients eligible for the study were unwilling to participate.

Indication to operate generally follows a defined preoperative diagnostic protocol including standardised history questionnaires, physical and proctological examination, anorectal physiological testing (anal monometry, balloon expulsion test) and diagnostic imaging (video-defecography, contrast enema).

Within this routine all patients are asked to fill in the “International Consultation on Incontinence Questionnaire – Short Form “ (ICIQ-SF), a validated tool for the assessment of urinary symptoms [9–11]. This questionnaire is supplemented by a “quality of life “ (QoL) visual analogue scale (0 to 10) which is not part of the ICIQ-SF and graded inversely to the score.

Also within the framework of our usual preoperative assessment, patients routinely have a sonographic measurement of residual urine. Overall, the study did not alter our usual preoperative routine.

A control examination (clinical assessment, scores) is also routinely scheduled for all patients at 6 to 8 weeks postoperatively. In addition to the usual postoperative routine, patients participating in this study were asked to again fill in the ICIQ-SF questionnaire as well as a customised study questionnaire and undergo a repeat ultrasound assessment of residual urine.

For the study, post-void residual urine measurements of less than 100 ml were considered insignificant and were considered as “unchanged”. Likewise, a difference of less than 3 points between pre- and postoperative scores was considered as “unchanged”.

### Statistical analysis

Since there are no data permitting a valid sample size calculation, a “convenience” sample size of 100 patients was chosen.

Patient characteristics were summarised using descriptive statistics. Differences between pre- and postoperative variables were tested with the paired Wilcoxon test with continuity correction. *P* < 0.05 was considered statistically significant. The statistical analysis was performed using the statistical software R version 4.0.2 (R Core Team 2020) [12].

### Ethics approval

This study was approved by the local ethics board (2015–327-f-S, Ethikkommission der Ärztekammer Westfalen-Lippe) and registered as a clinical study with the German national database (DRKS00010207, Deutsches Register Klinische Studien).

## Results

### Preoperative details

Table 1 summarises patients demographics, previous operations and obstetric history. The majority of patients reported constipation as their chief complaint (59%) or a combination of constipation and incontinence (27%) (Table 2); this is also reflected by the respective scores (Table 2). Relevant preoperative clinical and radiological findings are summarised in Table 3. Defecography showed significant anterior rectocele formation of ≥ 2 cm [13] in 85% of patients; 89% of patients were graded Oxford III–V, thereby documenting increasing degrees of rectoanal intussusception or external rectal prolapse [13]. Table 4 summarises the preoperative functional assessment, revealing abnormal balloon expulsion tests and anal manometry in the majority of patients.

**Table 1:** Demographics

**Demographics (*n*= 100)**	
***Age (years: median, range)***	54 (16–78)
***Previous operations (patients)***	90
Hysterectomy	31
Gyn other	5
Pelvic floor/bladder	6
Proctology	14
Major abdominal	6
Minor abdominal	51
***Obstetric history***	
**Vaginal deliveries**	
0	26
1	16
2	30
3 +	17
n.a	11
**Obstetric injury**	
Episiotomies, perineal tears	36
Deliveries by forceps, suction cup	6
No trauma	47
n.a	11

*n.a.* not available

**Table 2:** Preoperative symptoms and scores

Pre-op symptoms and scores*	
***Leading abdominal complaint***	
Constipation	59
Incontinence	4
Both	27
n.a	10
***Constipation score (0–30)***	8 (0–23)
n.a	20
***Incontinence score (0–20)***	3 (0–11)
n.a	21
***Proctological symptom scale (0–40)***[24]	5 (0–36)
n.a	24

*  n.a.* not available

^*^score points, median, range

**Table 3:** Preoperative clinical and radiological findings

**Examination (*n*** **= 100)**	*n* (= %)	
***Descending perineum (DP)***	45	
* Dynamic (on straining only)*	*28*	
* Fixed (at rest)*	*7*	
* Not detailed*	*10*	
No DP	24	
n.a	31	
***Anterior rectocele (AR)***	62	
No AR	17	
n.a	21	
***Rectal prolapse***		
Occult grade II–III	71	
Complete	9	
n.a	20	
**Contrast enema (***n* **= 97)**	*n*	%
***Diverticulosis***	62	64
***Colon, sigmoid elongation***	89	92
**Video defaecography (***n* **= 87)**		
***Rectal intussusception (Oxford grading)***	*n*	%
Grade I	1	1
Grade II	3	3
Grade III	15	17
Grade IV	56	64
Grade V	7	8
Not determinable	5	6
***Sigmoidocele***	86	99
No sigmoidozele	1	1
***Anterior rectocele***	85	98
Up to 2 cm	11	13
> 2–3 cm	25	29
> 3–4 cm	29	33
> 4 cm	20	23
No rectocele	2	2
***Posterior Rectocele***	36	41
Perineal	28	32
“High” (puborectal)	8	9
***Descending Perineum (DP)***	86	99
Grade I	8	9
Grade II	8	9
Grade III	70	80
No DP	1	1

*n.a.* not available

**Table 4:** Preoperative functional assessment

**Balloon expulsion test (*n*** **= 86)**		
***Perception***	*n*	%
Normal (up to 30 ml)	40	47
Slightly reduced (up to 50 ml)	35	41
Moderately reduced (up to 80 ml)	5	6
Severely reduced (> 80 ml)	6	7
***Defecation urge***		
Normal (up to 50 ml)	19	22
Reduced (up to 100 ml)	41	48
Severely reduced (> 100 ml)	26	30
***Max. tolerated volume***		
Normal (up to 100 ml)	22	26
Moderately increased (up to 200 ml)	49	57
Severely increased (> 200 ml)	15	17
***Balloon expulsion***		
Normal (spontaneous)	16	19
* Traction required*		
Up to 100 g	10	12
Up to 200 g	13	15
Up to 350 g	4	5
Negative at 350 g	43	50
**Anal manometry (*n* = 71)**		
***Resting pressure***	*n*	%
Normal (> 40 mm Hg)	54	76
Low	17	24
***Max. squeeze pressure***		
Normal (> 80 mm Hg)	47	66
Low	24	34
***Resting and squeeze pressure low***	12	17
***Tmax squeeze (seconds)***		
Not measureable ≤ 2 s	56	79
Normal (> 2 s)	15	21

### Operative treatment

Operative details are listed on Table 5. Resection was extended to left hemicolectomy in eleven patients due to coexisting advanced diverticular disease. Minor (Clavien–Dindo grade II) and major (Clavien–Dindo Grade IIIb) operative complications were recorded in 4% and 6%, respectively. Median hospital stay was 7 days.

**Table 5:** Operative treatment

**Laparoscopic resection rectopexy (*n* = 100)**	*n* (= %)
***Extent of resection***	
Sigmoid resection	89
Left hemicolectomy	11
***Mobilisation of rectum***	
Circular	74
Dorsal and lateral	26
Conversion	1
Protective ileostomy	1
**Complications (Clavien–Dindo)**	
***Grade II***	***4***
Urinary infection	3
Wound infection	1
***Grade IIIb***	***6***
Anastomotic leakage	4
Injury to left ureter	1
Anastomotic bleeding	1
**Postoperative hospital stay**	Days
Median	7
Range	6–43

### Urinary symptoms

At their 6- to 8-week postoperative visit, 24% of patients reported an improvement of their urinary function, against 14% who noticed an increase of urinary complaints (Table 6).

**Table 6:** Post-op change in urinary function

**Questionnaire: has your urinary function changed? (6–8 weeks post-op)** *n* = 100 (= %)				
***Noticed change of***		**Continence**	**Frequency**	**Voiding**
*Moderately improved*	11	9	4	5
*Much improved*	13	10	6	9
*Moderately worse*	10	4	3	7
*Much worse*	4	2	4	4
*Urinary function unchanged*	62			

Crosschecking “symptom memory” by comparing preoperative ICIQ scores with questioning (“did you suffer urinary symptoms prior to the operation?”) at the postoperative visit revealed that 6% of patients reported having had urinary symptoms before the operation in contradiction to their negative (i.e. “0 “) preoperative scores.

An overall highly significant postoperative increase in QoL scoring was noted for the entire collective of patients. The QoL scale is an addition to the ICIQ-SF questionnaire which is assessed separately. The scaling is inverse to the scoring: the higher the scale value, the better is the QoL; the higher the score, the more the urinary symptoms (Table 7).

**Table 7:** Comparison pre- and postoperative QoL and ICIQ

**QoL (all patients,** ***n*** **= 100)**		
	**Pre-op**	**Post-op**
	*(median / range)*	
***QoL (0–10)***	5/0–10	7/0–10
**Pre- to postoperative QoL**	*p* < 0.001*	
**ICIQ (all patients, *n* = 100)**		
	**Pre-op**	**Post-op**
	*(Median/range)*	
***Frequency (0–5)***	0/0–5	0/0–5
***Quantity (0–6)***	0/0–4	0/0–4
***Impairment (0–10)***	0/0–10	0/0–10
**ICIQ total (0–21)**	0/0–18	0/0–18
**Pre- to postoperative ICIQ**	*p* < 0.05*	
**Subgroup analysis: pre-op positive ICIQ “ICIQ-symptomatic patients” (*n* = 45)**		
	**Pre-op**	**Post-op**
	*(Median/range)*	
***Frequency (0–5)***	2/1–5	1/0–5
***Quantity (0–6)***	2/2–4	2/0–4
***Impairment (0–10)***	3/0–10	1/0–10
**ICIQ total (0–21)**	7/3–18	4/0–18
**Pre- to postoperative ICIQ**	*p* < 0.001*	

^*^Paired Wilcoxon test with continuity correction

Also, pertaining to the entire collective, postoperative ICIQ scoring was significantly improved (Table 7). With the exception of five patients experiencing “de novo” symptoms (see below), these effects were exclusively noted among patients with preoperative positive ICIQ scores (i.e. those having reported preoperative urinary symptoms). This is not entirely surprising: patients with negative ICIQ scores (i.e. “0” = “no urinary symptoms”) cannot improve their postoperative ICIQ scoring. In consequence, the proportion of asymptomatic patients dilutes any potential effects on urinary symptoms caused by the operation.

The 45 patients who had “positive” preoperative scores were therefore subanalysed. In this “undiluted” subgroup, a highly significant decrease of scoring was confirmed, this indicating ICIQ-relevant improvement of urinary function (Table 7).

Table 9 presents a more detailed analysis of the pre- to postoperative migration in the ICIQ scores. Overall, 19% of patients scored “better” postoperatively (defined as a decrease of 3 or more score points). In contrast, 8 patients scored worse. Within this subgroup of 8 patients, 5 had had “zero” scores preoperatively, indicating that these 5 patients experienced “de novo” symptoms postoperatively (Table 8). Overall, pre- and postoperative ICIQ scoring correlated in 81% with the responses patients gave when asked whether they experienced a postoperative change in bladder function (Table 9).

**Table 8:** Migration of pre- to postoperative ICIQ scores

**Change of pre- and post-op ICIQ scores (*n*** **= 100 = %)**		
**No change**	**73**	
Pre-op “0 “ to post-op “0 “ (pre- and post-op numerical score negative)	50	
Pre- and post-op numerical score positive but unchanged ***(*± *2 score points)***	23	
		**Score change** ***Median/range***
**ICIQ post-op “better” *(numerical minus of 3 or more score points)***	**19**	5 (3–13)
Pre-op numerical score positive change to “0 “ (negative)	14	
Post-op numerical score improved	5	
**ICIQ post-op “worse “ *(numerical plus of 3 or more score points)***	**8**	5 (3–15)
“De novo” symptoms: pre-op “0 “ (negative) to post-op positive score	5	
Score worse	3

**Table 9:** Correlation of pre- and post-op ICIQ scoring and symptom reporting

**Correlation of change of ICIQ scoring and questionnaire reporting of “change of urinary symptoms”**			
*n* = 100 (= %)			
	**n**	***Subjective experience***	***n***
***Pre- and post-op ICIQ negative***	**50**	Bladder function unchanged	41*
		Bladder function improved	4
		Bladder function worse	5
***ICIQ unchanged (*± *2 score points)***	**23**	Bladder function unchanged	18*
		Bladder function improved	2
		Bladder function worse	3
***Post-op decrease of ICIQ (*> *2 score points)***	**19**	Bladder function unchanged	3
		Bladder function improved	16*
		Bladder function worse	0
***Post-op increase of ICIQ (*> *2 score points)***	**8**	Bladder function unchanged	0
		Bladder function improved	2
		Bladder function worse	6*

^*^Correlation of ICIQ scoring and symptom reporting: 81%

On the other hand, the comparison of pre- and postoperative residual urine measurements (RUM) remained inconclusive, as pre- and postoperative measurements in all patients tested was less than 100 ml (pre-op (*n* = 92), 0–90 ml, median 0 ml; post-op (*n* = 87), 0–90 ml, median 0 ml).

## Discussion

Rectal prolapse is rarely an isolated phenomenon. More commonly, it is a partial aspect of an acquired degenerative process involving the pelvic floor as a whole [14]. Hallmarks of this process are progressive descent and relative topographic alterations of pelvic organs leading to distortion of pelvic anatomy and secondary micromorphologic tissue damage [15]. As the rectum descends and intussuscepts, the bladder commonly also descends caudally (and vice versa). All this furthers obstruction and other deficits affecting pelvic floor function, foremost micturition and defecation. In addition to bowel-related problems, as many as 50% [16] of female patients presenting with rectal prolapse therefore also report urinary symptoms such as voiding problems and urinary incontinence. In our collective, 45% of patients recorded a positive preoperative ICIQ score indicating coexisting urinary symptoms (Table 8).

Conversely, detailed interviews of female patients seeking therapy for their functional bladder disorders commonly reveal concomitant symptoms of obstructed defaecation or anal incontinence in up to 80% [17, 18]. A recognition that morphological changes and functional deficits of pelvic floor dysfunction are usually not limited to just one organ entity is the rationale behind interdisciplinary “pelvic floor centres” which have meanwhile been established in many parts [19–21].

The principles of laparoscopic resection rectopexy are complete mobilisation of the rectum down to the pelvic floor. In addition, the pendent sigmoid colon is resected, as to elevate and straighten the entire left-sided colorectum up towards the left colonic flexure, without any significant residual looping. It may be speculated that as a side effect of this procedure, the straightening of the mobilised rectum at least partially also elevates the ventral compartment including the bladder, thereby potentially correcting any preexisting descent and improving bladder function. On the other hand, deep mobilisation of the rectum may cause temporary or permanent operative trauma to the autonomic nerve supply, thereby impairing bladder function [22]. So far, neither of the aforementioned potential effects of this procedure have been systematically assessed.

This study concludes that laparoscopic resection rectopexy does have highly significant effects on preexisting urinary symptoms. Following this procedure, 19 (42%) of the 49 “ICIQ-symptomatic “ patients (those with numerically positive preoperative ICIQ-SF) scored less (“better “) by three or more points postoperatively. Among these 19 patients, 14 were even rendered “ICIQ symptom-free” (postoperatively scoring “0 “). Conversely, to a lesser degree, there was also symptomatic deterioration: three patients (7% of the 49 preoperative ICIQ-symptomatic patients) scored three or more points higher (“worse “) postoperatively. In addition, five patients among the 49 in this subgroup experienced “de novo” symptoms after the operation, scoring positively in their postoperative assessment.

These data have to be viewed with some caution. There generally remains an unavoidable and therefore accepted level of uncertainty when interpreting scores [23]. This is confirmed also by this study. In 19% of patients, the stated subjective experience of urinary function before or after the operation, when asked directly face-to-face, did not correlate to the associated ICIQ-SF scoring done prior to the interview.

A further discrepancy was detected when patients were asked (at their postoperative visit) if they could remember having had urinary symptoms prior to the operation: six patients reported remembering such symptoms, although their preoperative ICIQ-SF scores were “0”.

When patients were asked to clearly state whether their bladder function had changed following the operation, 24% reported a better function (against 19% who had improved score values), 14% reported deterioration of their bladder function (against 8% scoring accordingly).

Despite these limitations, the ICIQ-SF can still be considered a simple and sufficiently reliable screening tool for urinary symptoms in most patients. This is in contrast to residual urine measurements which did not sufficiently correlate to symptoms to be of any meaningful value for the purpose of this study.

This study has shown that the lifting repair of the posterior compartment of the pelvic floor by laparoscopic resection rectopexy can have beneficial or, to a lesser extent, detrimental effects on the anterior compartment, bladder function in particular. So far, it is not possible to predict effects on an individual basis. It appears probable that irrespective of the ongoing technical debate, similar effects may be found for most alternative operative procedures for the treatment of rectal prolapse. It is surprising that in this era of “pelvic floor centres”, we still know so little about such “collateral effects” considering the broad armamentarium of pelvic repair procedures which are done routinely in great numbers. Without more factual knowledge and awareness about these effects, expert guidance of patients appears limited. In future studies, these potential effects should therefore be considered and further investigated, irrespective of the operative method.
